# Do symptoms and signs of temporomandibular disorders have an association with breathing pattern: a cross-sectional study on Turkish children and adolescents

**DOI:** 10.1186/s12903-024-04482-5

**Published:** 2024-06-24

**Authors:** Mehmed Taha Alpaydin, Tugce Alpaydin, Damla Torul

**Affiliations:** 1 Department of Orthodontics, Faculty of Dentistry, Ordu University, Ordu, Turkey; 2 Department of Oral and Maxillofacial Surgery, Faculty of Dentistry, Ordu University, Ordu, Turkey

**Keywords:** Sleep apnea syndromes, Craniofacial abnormalities, Pediatric

## Abstract

**Background:**

This paper aimed to explore the prevalence of temporomandibular disorders (TMDs) signs/symptoms, and to investigate the possible link between signs/symptoms of TMDs and mouth breathing (MB) by evaluating along with other risk factors, in a Turkish subpopulation of children and adolescence.

**Methods:**

This study was conducted with the archival data of the patients who applied with orthodontic complaints. Data on demographic characteristics, family-related factors, systemic status, occlusion, breathing patterns, oral habits, and bruxism were retrieved from the archival records.

**Results:**

Nine hundred forty-five children and adolescents with a mean age of 14.82 ± 2.06 years were included in the study. Of the participants, 66% were girls, 60.4% were delivered by C-section, 8.4% of the participants had at least one systemic disease, 9.2% of the participants had allergy, and 4.3% of the participants’ parents were divorced, 18.7% have an oral habit, 6.6% have bruxism, 29.8% have malocclusion and 14.1% have MB. Eight-point-five percent of participants have signs/symptoms of TMD. Among them 2.9% have pain, 3.7% have joint sounds, 1.4% have deflection, and 3.9% have deviation. Evaluation of the risk factors revealed a significant relation between the signs/symptoms of TMD and bruxism (OR 8.07 95% CI 4.36–14.92), gender (OR 2.01 95% CI 1.13–3.59), marital status of parents (OR 2.62 95% CI 1.07–6.42), and MB (OR 3.26 95% CI 1.86–5.71).

**Conclusions:**

According to the study’s findings, girls and those with bruxism, divorced parents, and MB behavior are more likely to have signs/symptoms of TMD. Age found to have significant effect on the occurrence of the signs/symptoms of TMD alone, but together with other factors the effect of the age is disappeared. Early screening and intervention of MB as well as the signs/symptoms of TMD can help to limit detrimental effects of these conditions on growth, and quality of life of children and adolescents.

## Background

Temporomandibular disorders (TMDs) is a term referring to impairments related to masticatory muscles, the temporomandibular joint (TMJ), and adjacent structures [1, 2]. Signs/symptoms of TMDs include pain, restriction or deviation/ deflection in mouth opening and sounds [3]. Children may exhibit TMD signs/symptoms, which gradually worsen as they approach adolescence. TMDs are reported to affect between 20% and 60% of adolescents and children, with a female preponderance [1, 4, 5]. The etiology of TMDs is not elucidated and is considered as multifactorial. Oral habits, bruxism, psychosocial factors, systemic status, sociodemographic characteristics, malocclusion, postural changes, sleep quality, satisfaction with life, breathing patterns, and family factors are among the factors reported to contribute to the occurrence and perpetuation of TMDs [6–13].

Mouth breathing (MB) is one of the most prevalent and underappreciated symptoms of sleep-disordered breathing (SDB) which includes snoring, upper airway resistance syndrome (UARS), and obstructive sleep apnea (OSA) [14]. MB is described as a condition in which the correct nasal breathing pattern is replaced by supplemental MB or mixed breathing for more than six months [15, 16]. The prevalence of MB is estimated approximately 11–56% in children and adolescents [17, 18]. MB may originate from genetic factors, improper growth of maxilla associated with malocclusions and/or oral clefts, and nasal obstruction because of enlarged adenoids or tonsils, allergic rhinitis, and deviation of the nasal septum or indirectly from deleterious oral habits [16, 19–22]. MB may disappear with age related shrinkage of adenoids [23–25]. Otherwise, it will have a permanent negative impact on the child’s teeth and facial development [17]. Also, related to neural adaptations, persistent MB can be perpetuated into adulthood and changes the control of the upper airway and the muscular function [16].

Normal nasal breathing, according to Moss’s functional matrix theory, favors healthy growth and development of the craniofacial structures by providing appropriate interaction between mastication, swallowing, and other components [18, 19]. By affecting the developmental process MB can result in craniofacial and dental disturbances, including long face-“adenoid faces”, narrow nostrils, class II malocclusion, maxillary hypoplasia, posterior crossbite, posture alteration, gingivitis, and dental decay [18, 19, 26]. Moreover, MB causes immature auditory processing, fatigue, restless sleep, nocturnal enuresis, failure to thrive, inadequate oxygenation of the brain, learning impairments and has a negative effect on the daily activities [15, 27].

MB is considered to cause growth disturbance in the structures of the craniofacial complex [17, 19, 26, 28]. Recent experimental studies suggested that MB impairs the development of TMJ in early stages [18, 29]. Based on these, the authors hypothesized that individuals with MB may tend to have TMD signs/symptoms more commonly and there can be a link between MB and TMD. Therefore, this paper aims to explore the prevalence of TMD signs/symptoms and to investigate the possible link between signs/symptoms of TMD and MB by evaluating along with other risk factors, in a subpopulation of Turkish children and adolescents.

## Methods

### Study participants

This retrospective study was conducted with the archival data of the patients who applied for the Department of Orthodontics of Ordu University with orthodontic complaints. The study protocol was approved by the Ethics Committee of the Ordu University (No:2023/208) and was conducted following the ethical standards specified in the Helsinki Declaration.

The inclusion criteria were as follows:

1. Patients under the age of 18
2. Patients not having any syndrome in the maxillofacial region
3. Patients not having a history of operation in the head and neck region

The exclusion criteria were as follows:

1. Patients who have missing archival data

### Clinical parameters evaluated

Clinical examination was performed by the clinicians who are familiar with TMD.

Sociodemographic data: Age and gender of the patients, number of siblings, and marital status of the parents (Married/Divorced) were recorded.

Systemic disease/ Presence of allergy: Recorded based archival data as present or absent.

Sagittal malocclusion type: Sagittal malocclusion recorded as Angle Class I, Class II, Class III.

Transversal malocclusion type: Posterior and anterior cross bite were recorded as present or absent, based on archival data.

Bruxism: Recorded as present/absent based on archival data. Bruxism has been evaluated in clinical examination by considering the patient’s history, clinical examination, and tooth wear [30].

Oral habits: Finger or lip sucking, and tongue thrust recorded based on the patient/parent reports as present or absent.

Breathing patterns: This was determined during examination by asking the patient to close his/her mouth for 30 s, and the mirror was placed under the right and left nostrils. The mist was observed in the mirror. If the mist is observed in the mirror, there is nasal breathing, if not observed, oral breathing is recorded as present.

Birth Delivery: Recorded based on the parent’s report and classified as cesarean section or normal birth delivery.

TMD: Recorded based on the presence of at least one sign/symptom (sounds, pain, deflection/deviation, and limitation in mouth opening) as present or absent.

TMJ sounds: Clicking, popping, and crepitus were evaluated by using a stethoscope.

Pain in TMJ: Pain in the TMJ area on palpation or mandibular movement.

Pain in the muscles: Pain on palpation of the masticatory muscles.

Deviation/deflection: Based on the clinical observation of the presence of deviation/deflection during mouth opening.

The maximum mouth opening (MMO): Distance measured between the incisal edges of the right 1st maxillary and mandibular central incisors.

### Statistical analysis

The data were analyzed using Statistical Package for the Social Sciences (version 20.0, IBM Corp, Armonk, NY). Categorical variables are given as numbers/percentages and continuous variables are presented as mean ± standard deviation. Chi-square analysis was used to explore the relation between the TMD and categorical variables. Normality was evaluated with the Kolmogorov-Smirnov test. For the comparison of the continuous variables between patients with/without TMD Mann-Whitney U test was used. Regression analysis was performed for advanced analysis of the variables. Univariate analysis was performed first and multivariate analysis was conducted with the parameters which found statistically significant in univariate analysis. A P value < 0.05 was accepted as significant with a 95% confidence interval (CI).

## Results

### Study participants

Nine hundred forty-five children and adolescents were included in the study. Participants’ mean age was 14.82 ± 2.06 years, and 66% of the participants were girls. The mean age of girls was 14.90 ± 2.02 and male was 14.80 ± 2.14 years. Of the participants, 60.4% were delivered by C-section, 8.4% of the participants had at least one systemic disease, 9.2% of the participants had allergies, and 4.3% of the participants’ parents were divorced. The mean number of siblings was 2.25 ± 0.96. No significant difference was observed between participants with or without TMD regarding the number of siblings (*p* = 0.726), systemic status (*p* = 0.895), allergy (*p* = 0.509), and delivery type (*p* = 0.176), while significant differences were seen regarding gender (*p* = 0.024), age (*p* = 0.008), and marital status of the parents (*p* = 0.009). The demographic data of the participants are shown in Table 1.

**Table 1:** Descriptive characteristics of the participants

		Total		No TMD		TMD	
		*n*	%	*n*	%	*n*	%
Gender	Girl	624	66	562	65	62	77.5
	Boy	321	33	303	35	18	22.5
Marital status of parents	Married	904	95.7	832	96.2	72	90
	Divorced	41	4.3	33	3.8	8	10
Delivery Type	Normal	374	39.6	348	40.2	40	38.1
	C-section	571	60.4	517	59.8	65	61.9
Systemic disease	No	866	91.6	793	91.7	73	91.3
	Yes	79	8.4	72	8.3	7	8.8
Allergy	No	858	90.8	787	91	71	88.8
	Yes	87	9.2	78	9	9	11.3
Breathing pattern	Nose	812	85.9	758	87.6	54	67.5
	Mouth	133	14.1	107	12.4	26	32.5
Oral habits	No	768	81.3	709	82	59	73.8
	Yes	177	18.7	156	18	21	26.3
Bruxism	No	883	93.4	827	95.6	56	70
	Yes	62	6.6	38	4.4	24	30
Sagittal	1	282	29.8	266	30.8	16	20
	2	481	50.9	436	50.4	45	56.3
	3	182	19.3	163	18.8	19	23.8
Anterior crossbite	No	857	90.7	783	90.5	74	92.5
	Yes	88	9.3	82	9.5	6	7.5
Posterior crossbite	No	822	87	755	87.3	67	83.8
	Yes	123	13	110	12.7	13	16.3
		**Mean**	**Std.** **Deviation**	**Mean**	**Std.** **Deviation**	**Mean**	**Std.** **Deviation**
Age of children and adolescence		14.82	2.06	14.77	2.07	15.36	1.91
Number of Siblings		2.25	0.96	2.25	0.96	2.25	0.97

### Presence of TMD signs/symptoms

Among the 945 participants, 8.5% have signs/symptoms of TMD. Twenty-seven (2.9%) have TMJ or muscle pain, 35 (3.7%) have TMJ sounds, 13 (1.4%) have deflection, and 37 (3.9%) have deviation. The mean MMO among the participants was 44.35 ± 5.26 mm. Regarding MMO no significant difference was found between patients with/without TMD (*p* = 0.321).

### Bruxism

A total of 6.6% of the participants had bruxism. Thirty percent of the participants with signs/symptoms of TMD had bruxism while 4.4% of the participants without signs/symptoms of TMD had bruxism. A significant difference was seen regarding bruxism (*p* < 0.001) between patients with or without TMD.

### Oral habits

**Fig. 1:**
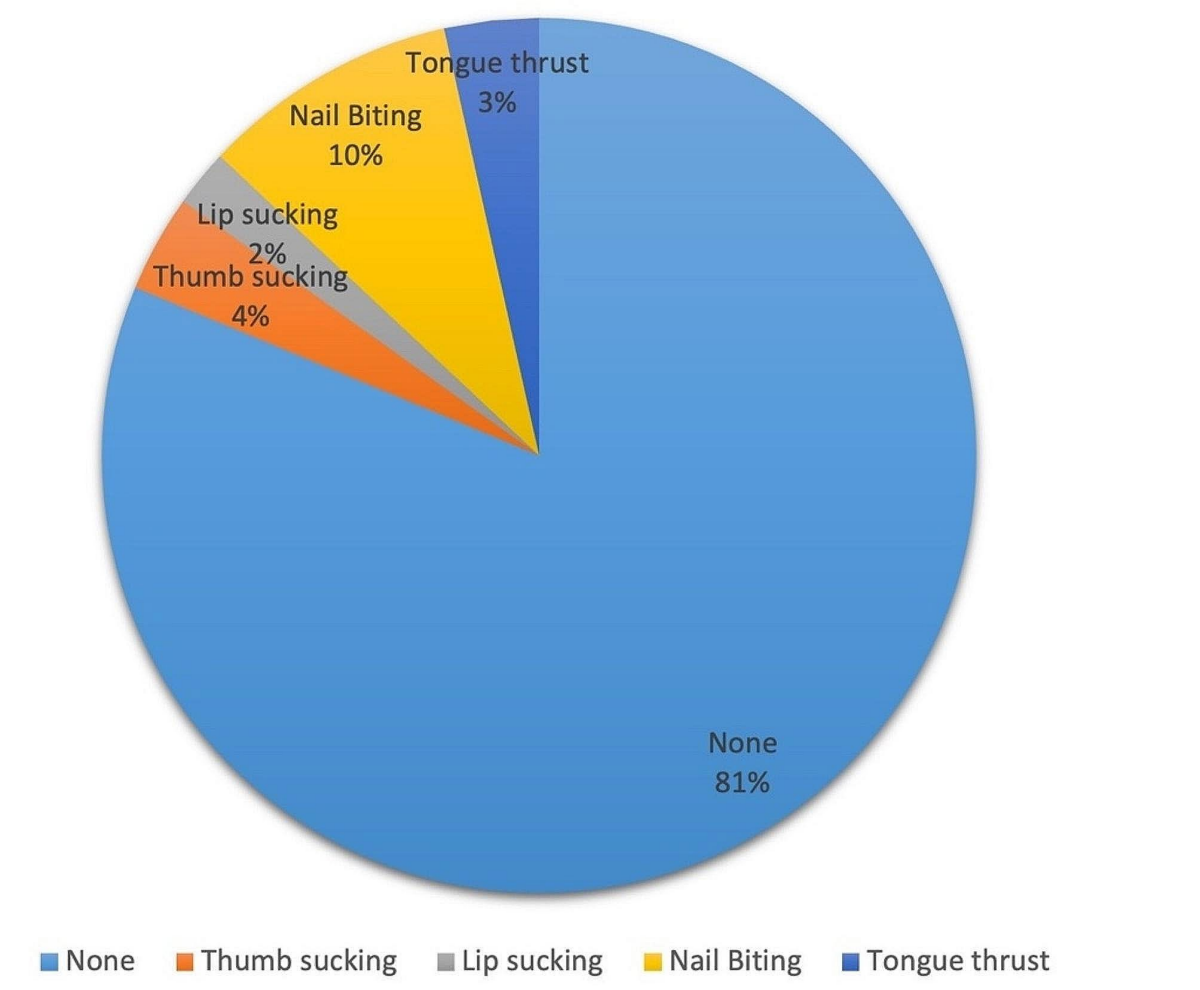
Oral habit distribution of individuals

A total of 18.7% of the participants had oral habits (at least one). 26.3% of the participants who have signs/symptoms of TMD, also have at least one oral habit while 18% of the participants who did not have signs/symptoms of TMD have at least one oral habit (Fig. 1). No significant difference was seen regarding oral habits (*p* = 0.072) between participants with or without TMD.

### Malocclusion

All the participants had at least one type of malocclusion. Regarding sagittal malocclusion 29.8% of the participants have Class I, 50.9% have Class II and 19.3% have Class III malocclusion. Among the participants who have signs/symptoms of TMD, 20% have Class I, 56.3% have Class II and 23.8% have Class III malocclusion. Among the participants who did not have signs/symptoms of TMD, 30.8% have Class I, 50.4% have Class II and 18.8% have Class III malocclusion. No significant difference was found between participants with/without TMD for sagittal malocclusion (*p* = 0.120).

Regarding anterior cross bite a total of 9.3% of the participants were detected to have. In 7.5% of the participants who have and 9.5% of the participants who did not have TMD signs/symptoms, anterior crossbites were observed. No significant difference was detected between participants with/without TMD regarding anterior crossbite (*p* = 0.560).

Regarding posterior cross bite a total of 13% of the participants were detected to have. In 16.3% of the participants who have and 12.7% of the participants who did not have TMD signs/symptoms, posterior cross bites were observed. No significant difference was seen between participants with/without TMD regarding posterior crossbite (*p* = 0.369).

### Breathing Pattern

Regarding breathing patterns, a total of 14.1% of the participants were detected to have MB. 32.5% of the participants who have and 12.4% of the participants who did not have TMD symptoms detected to have MB. A significant difference was seen regarding breathing patterns (*p* < 0.001) between participants with or without TMD.

### Results of logistic regression analyses

The presence of TMD signs/symptoms was found to be associated with gender, age, bruxism, marital status of the parents, and breathing pattern in univariate analysis (*p* < 0.05). Multivariate analyses revealed that gender, bruxism, marital status of the parents, and breathing pattern were observed to increase the risk for TMD signs/symptoms. The risk of having signs/symptoms of TMD for the female gender was 2.01 (95% CI 1.13–3.59) fold greater than males. The risk of having signs/symptoms of TMD was found to be increased 2.62 (95% CI 1.07–6.42) fold in the participants who have divorced parents. The risk of having signs/symptoms of TMD is seen to be increased by 3.26 (95% CI 1.86–5.71) fold in mouth breathers compared to the nasal breathers. The risk of having signs/symptoms of TMD was detected to be increased by 8.07 (95% CI 4.36–14.92) fold in the presence of bruxism (Table 2).

**Table 2:** Univariate and multivariate logistic regression models of risk factors for TMD (*N* = 945)

		*N*	*n* (%)	Crude OR	95% Cl		*p*	Adjusted OR	95% Cl		*p*
Age of children and adolescent				1.17	1.04	1.32	**0.006**	1.12	0.99	1.27	0.071
Gender	Girl	624	62	1.85	1.07	3.19	**0.025**	**2.01**	**1.13**	**3.59**	**0.017**
	Boy(ref)	321	18								
Marital status of parents	Married (ref)	904	72								
	Divorced	41	8	2.8	1.24	6.29	**0.013**	**2.62**	**1.07**	**6.42**	**0.035**
Number of siblings				0.99	0.78	1.26	0.977				
Delivery type	Normal (ref)	374	40								
	C-Section	571	65	1.39	0.85	2.27	0.178				
Systemic diseases	No (ref)	866	73								
	Yes	79	7	1.05	0.46	2.37	0.895				
Allergy	No (ref)	858	71								
	Yes	87	9	1.27	0.61	2.65	0.510				
Breathing pattern	Nose (ref)	812	54								
	Mouth	133	26	3.41	2.04	5.67	**< 0.001**	**3.26**	**1.86**	**5.71**	**< 0.001**
Bruxism	No (ref)	883	59								
	Yes	62	21	9.32	5.23	16.63	**< 0.001**	**8.07**	**4.36**	**14.92**	**< 0.001**
Oral habit	No (ref)	768	56								
	Yes	177	24	1.61	0.95	2.74	0.074				
Sagittal	1 (ref)	282	16								
	2	481	45	1.71	0.95	3.09	0.073				
	3	182	19	1.93	0.96	3.87	0.061				
Anterior crossbite	No (ref)	857	74								
	Yes	88	6	0.77	0.32	1.83	0.561				
Posterior crossbite	No (ref)	822	67								
	Yes	123	13	1.33	0.71	2.49	0.370				

**ref**: reference category, **Bold characters**: Significant risk factors in univariate and multivariate analysis

## Discussion

TMDs are a public health problem in the pediatric population in terms of their effect on the growth of craniofacial structures and several affected subjects [2, 3]. Unfortunately, awareness regarding these consequences is low, and little attention is paid to TMD in the pediatric population compared to the adults [3, 5]. Thus, this study aims to explore the prevalence of TMD signs/symptoms and to investigate the possible link between signs/symptoms of TMD and MB by evaluating along with other risk factors, in a subpopulation of Turkish children and adolescents. The results of this study showed that 8.5% of the participants have symptoms of TMD. This result is low when compared to the studies of Taneja et al. [31] in Indian (51%), Mora-Zuluaga et al. [32] in Colombian (71.5%), Tecco et al. [2] in Italian (44.1%), Stefano et al. [4] in Venezuelan-Italian (17.9-29.7%), Braido et al. [33] in Brazilian (16.2%), Mothgare et al. [34] in Indian (46%), Feteih et al. [35] in Arabian (21.3%), Khan et al. [36] in Brazilian-Canadian-French (31.6%, 23.4%, 31.8%) populations and similar to the study of Ak et al. [37] (9%) in the Turkish population. Regarding the signs/symptoms, deviation (3.9%) is the most frequent symptom followed by TMJ sounds (3.7%), pain (2.9%), and deflection (1.4%). In the study of Mora-Zuluaga et al. [32] the most common symptom was unilateral sound (33.8%), followed by pain (26%). Similarly, Feteih et al. [35] reported joint sounds as the most frequent sign (13.5%) followed by restricted mouth opening (4.7%) and deviation (3.9%). Rauch et al. [6] found the most prevalent symptoms as headache (55.7%) and joint sounds (17.6%). There is a wide range of variations regarding the results of the studies conducted in children and adolescents. This may originate from the differences in terms of sample sizes, age groups, ethnic origins, and the methods of TMJ evaluation in the studies.

Age and gender have long been investigated parameters regarding TMD in the literature. A general thought is that the TMD tends to be high in girls. In most of the studies, TMDs were most prevalent among girls compared to the boys [4, 6, 34–36, 38]. However, some studies have not found this difference between the two genders [33, 39, 40]. Similar to the studies favoring girl dominance in TMD, in the present study 66% of the participants were constituted of girls. Also, a 2.1-fold increased risk of having signs/symptoms of TMD was found for the girls compared to the boys. This result is also in concordance with the study of Khan et al. [36] reported a 1.96-fold increased risk regarding the girls.

The relationship between age and the prevalence of TMD in children and adolescents is still up for debate [39]. However, it is suggested that TMD tends to be higher with increasing age in children and adolescents. In terms of age, Tecco et al. [2] found that TMD was significantly more frequent in the age of 16–19 years (52.9%) compared to the age of 11–15 years (39.8%). Similarly, Jain et al. [8] suggested that as the age increases the presence of TMDs also increases. Kohler et al. [1] found in their longitudinal study that signs/symptoms of TMD such as clicking and locking were significantly higher in the older age group. They reported a 2.9 and 6.7-fold increased risk for clicking and locking respectively with the increasing age. In this study, a significantly increased risk of TMD was found in univariate analysis. This result may be originated from the increasing stress and decreasing adaptation mechanism of TMJ with age. However, significant affect of the age was not observed in multivariate analysis.

The systemic status of the participants was also investigated in terms of the effect on TMD in the studies. Kohler et al. [1] observed that participants receiving medical treatment had a 3.3-fold higher risk of having signs/symptoms of TMD. Also, the same researchers found that participants using regular drugs have a 5.5-fold higher risk of locking of the jaw [1]. Khan et al. [36] reported that painful TMD was related higher number of comorbidities and participants having allergies had a 1.43-fold higher risk of having painful TMD. Braido et al. [33] suggested a 2.5 and 3.1-fold higher risk of having painful TMD for the participants with bronchitis and asthma, respectively. In the present study, systemic status, allergy, and delivery type were investigated if they were risk factors for TMD. According to the results, none of them have a significant relation with TMD. This result may have originated from that the < 10% of the sample having allergy (9.2%) or systemic disease (8.4%). The factors related to lifestyle and family were also investigated as risk parameters for TMD. In Ostenjenso et al.’s [9] study living within a divorced family was found to significantly increase the likelihood of having TMD pain and also doing regular physical activity was observed to decrease the frequency of TMD. Pelkonen et al. [41] suggested that parent depression increases the risk of TMJ pain 1.81-fold. In this study, the risk of having signs/symptoms of TMD was found to be increased 2.62-fold in the participants who have divorced parents, however, no significant effect of the number of siblings on TMD was detected. This result may be the consequence of psychological distress on children and adolescents caused by the divorce of the parents. The authors think that increased attention and screening regarding TMD need to be done for children and adolescents who have divorced families. Awareness was also acquired among the families regarding TMD risk.

Oral habits are the commonly suggested risk factors for the occurrence of TMD and a high frequency of oral habits was reported in the studies previously. Perlman et al. [39] reported that 78.8% of the participants have at least one oral habit. Perrota et al. [42] reported oral habits in 21.3% of the participants. Motghare et al. [34] found nail biting (45.8%), and biting lips/objects (37%) of the participants as the most common habits and reported a significant relation between these habits, and signs/symptoms of TMD. Feteih et al [35]. observed lip/cheek biting was the frequent oral habit (41%), followed by nail-biting (29%), and thumb sucking (7.8%). In this study, a total of 18.7% of the participants had at least one oral habit and 26.3% of the participants who have signs/symptoms of TMD have oral habits. In regression analysis, however, oral habits were not found as a significant risk factor for TMD.

Imbalance in the stomatognathic system may result from the repetitive masticatory muscle activity known as bruxism, which is characterized by teeth clenching or grinding and/or bracing or thrusting of the mandible [37]. Ak et al. [37] and Feteih et al. [35] found the prevalence of sleep bruxism to be 60% and 7.4% in their studies. Kohler et al. [1] found to have a relation between clenching/grinding of teeth and TMD symptoms and signs. Motghare et al. [34], and Taneja et al. [31] observed a significant relationship between bruxism and signs/symptoms of TMD. Henrikson et al. [43] observed that tooth clenching increased the risk of TMD pain 27-fold. However, Khayat et al. [38] did not find a relation between bruxism and TMD. In the present study, a total of 6.6% of the participants have bruxism and 30% of the participants who have signs/symptoms of TMD also have bruxism. In multivariate analysis, bruxism was found to increase 8.07-fold having the risk of TMD signs/symptoms. This high-risk ratio may be due to the imbalance originating from the continuous load on the stomatogenic system in the bruxer subjects. This result reinforces the fact that as a commonly underestimated factor awareness regarding the detrimental consequences of bruxism needs to be provided to prevent the irreversible disturbances.

The relationship between malocclusion and TMD has been still debated and it seems that no consensus has been reached in the literature. Significant relationships with an overbite, cross-bite, deep bite, sagittal malocclusion, and transverse malocclusion were reported in different studies previously [8, 31, 32, 38, 40]. Tecco et al. [2] reported a 1.6-fold increased risk of having signs/symptoms of TMD in Class II malocclusion. Perrota et al. [42] found 2.25, 2.47, and 4.49-fold increased risk for TMD in unilateral and bilateral crossbite and negative overjet. Henrikson et al. [43] found that anterior open bite increases the risk of the presence of TMJ pain 3.9-fold and locking 5.7-fold. Mora-Zuluaga et al. [32] found that transverse malocclusion increase significantly the severity of TMD 12-fold. The results of the present study showed no significant difference between participants with or without TMD regarding sagittal malocclusion and anterior-posterior crossbite. Also, these variables were not found to a significant risk factors for having TMD signs/symptoms.

Nasal breathing in coordination with normal swallowing, mastication, and posture of the head, tongue, and lips promotes the proper growth and development of splanchnocranium [26]. MB has been associated with abnormal craniofacial growth including cranio-cervical hyperextension, speech alterations, upper airway obstruction and resistance, learning difficulties, and decreased quality of life [15]. Improvement in MB is linked to better behavior, less sleepiness, and an improvement in quality of life in children with MB and OSA, according to Bandyopadhyay et al. [15] is independent of improvement in OSA.

Disharmonized muscular function in MB leads to impairments in the development of craniomaxillofacial structures. In terms of these impairments, TMJ has some significance because the upper airway’s development is greatly influenced by the development of the TMJ, and the upper airway also has an impact on the TMJ’s health [29]. Wang et al. [18] reported that MB leads to cartilage layer thinning, subchondral bone resorption, and cartilage matrix destruction. Hu et al. [29] in their recent experimental study, found an early impact of MB on TMJ structures and suggested that early changes in MB behavior prevent the progression of MB-related alterations in the craniofacial complex. In the present study, 32.5% of the participants who have TMD were detected to have MB. The risk of having signs/symptoms of TMD was found to be increased 3.26-fold in the participants with MB. According to the results of this study, MB seems to increase the risk of having TMD. Since the facial skeletons grow and develop quickly, the “window for intervening” is limited. If MB is not treated during this time, it could have a long-lasting effect on how the face develops [17, 44]. By considering this, the result of this study reinforces the importance of awareness regarding MB behavior in the pediatric population and the necessity of early intervention and close screening.

In this study, the variables that may be related to TMD were assessed by controlling for these variables through regression analysis with an adequate sample size. This allows the authors to provide robust results and this is one of the strengths of this study. It is also important to note that there are several limitations regarding this study. Because of the cross-sectional design, causal and temporal relations among the variables cannot be inferred. We assessed the presence of variables such as systemic diseases, allergies, delivery type, bruxism and TMD through the archival records, so no definitive diagnosis was provided. The present study was conducted with the data obtained from a Turkish orthodontic subpopulation of children and adolescence which limits the generalizability of the results. We could provide only information about the presence of bruxism from the archival data so we could not provide information about nocturnal or diurnal bruxism. Also, we could not provide data about sleeping quality of the patients which is another limitation of this study.

## Conclusion

According to the study’s findings, girls and those with bruxism, divorced parents, and MB behavior are more likely to develop signs/symptoms of TMD. Age found to have significant effect on the occurrence of the signs/symptoms of TMD alone, but together with other factors the effect of the age is disappeared. Thus, it’s critical to pay closer attention to the early effects of MB on the craniofacial structure. Early screening and intervention of MB as well as the signs/symptoms of TMD is a necessity to provide normal development and function of craniofacial complex and can help to limit detrimental effects of these conditions on growth, and quality of life of children and adolescents. However, longitudinal studies are needed to clearly understand the causal and temporal relationship of TMD and MB.

## Data Availability

The datasets used and/or analyzed during the current study are available from the corresponding author on reasonable request.
